# Autochthonous Canine Pythiosis Linked with Freshwater Exposure, Italy, 2021–2024

**DOI:** 10.3201/eid3209.260354

**Published:** 2026-09

**Authors:** Andrea Peano, Anna Rita Molinar Min, Patrizia Danesi, Francesco Albanese, Nicla Furiani, Moira Monaco, Fabiano Necci, Chiara Noli, Sofia Sgubin, Antonella Varina, Mario Pasquetti

**Affiliations:** Universita degli Studi di Torino, Torino, Italy (A. Peano, A.R. Molinar Min, M. Pasquetti); Istituto Zooprofilattico Sperimentale delle Venezie, Legnaro, Italy (P. Danes, S. Sgubin); CDVet—Laboratorio Analisi Veterinarie, Rome, Italy (F. Albanese, F. Necci); Clinica Veterinaria Città dei Papi, Viterbo, Italy (N. Furiani); Veterinaria Cetego, Rome, Italy (M. Monaco); Ospedale Veterinario Anubi Bluvet, Ca’Zampa, Torino (C. Noli); private practice, Torino (A. Varina)

**Keywords:** pythiosis, fungi, *Pythium periculosum*, dog, Italy, lake, freshwater habitats, ecological niche, dermatology, skin, One Health

## Abstract

We report 7 recognized autochthonous cases of canine pythiosis identified in Italy during 2021–2024. All infected dogs had freshwater exposure. Cases involved multiple lakes and occurred over consecutive years. Sequenced isolates clustered within clade IV of the *Pythium insidiosum* complex, currently designated *Pythium periculosum*.

Pythiosis is a severe disease caused by pathogenic oomycetes of the genus *Pythium* and is classically associated with tropical and subtropical climates and freshwater environments (*1*,*2*). The disease affects mainly humans, horses, and dogs and is typically linked to exposure to stagnant or slow-moving freshwater habitats that support production of infective zoospores. Most mammalian infections involve the *Pythium insidiosum* complex, although there have been occasional reports citing additional pathogenic oomycetes (*3*,*4*).

Increasing reports from regions outside traditionally recognized endemic areas, including northern areas of the United States and Japan, suggest a broader global distribution of pythiosis (*2*). Europe, however, remains exceptionally affected. A recent systematic review identified Spain as the only country in Europe in which locally acquired human pythiosis cases had been documented (*2*,*5*). In 2023, we reported 2 autochthonous canine cutaneous pythiosis cases in Italy, which were linked to recurrent exposure to Lake Bracciano in central Italy (*6*). Those initial observations suggested the possible presence of a local ecologic niche favorable to pathogenic *Pythium* species. We subsequently identified 5 additional canine cases diagnosed during 2021–2024, bringing the total number of recognized locally acquired canine cases in Italy to 7. None of the dogs had traveled outside Italy before disease onset, supporting the notion of locally acquired infection.

All dogs had histories of repeated exposure to natural freshwater environments. Reported sites included Lake Bracciano (5 cases), Lake Bolsena (1 case), and Lake Viverone in northern Italy (1 case) (Figure 1). Distinct cases linked to Lake Bracciano were recognized from 2021 through 2024, suggesting persistent local environmental conditions favorable to pathogenic *Pythium* species. In addition, identification of a case associated with Lake Viverone expands the geographic range of recognized exposure sites by >300 km. Although environmental investigations were outside the scope of this study, all implicated sites feature shallow, near-shore areas that undergo marked seasonal warming during summer months. Such conditions may favor persistence or proliferation of pathogenic *Pythium* species (*1*). All factors considered, we believe that our findings support the presence of ecologic niches potentially suitable for pathogenic oomycetes in different regions of Italy despite the absence of traditionally recognized tropical climatic conditions.

**Figure 1:**
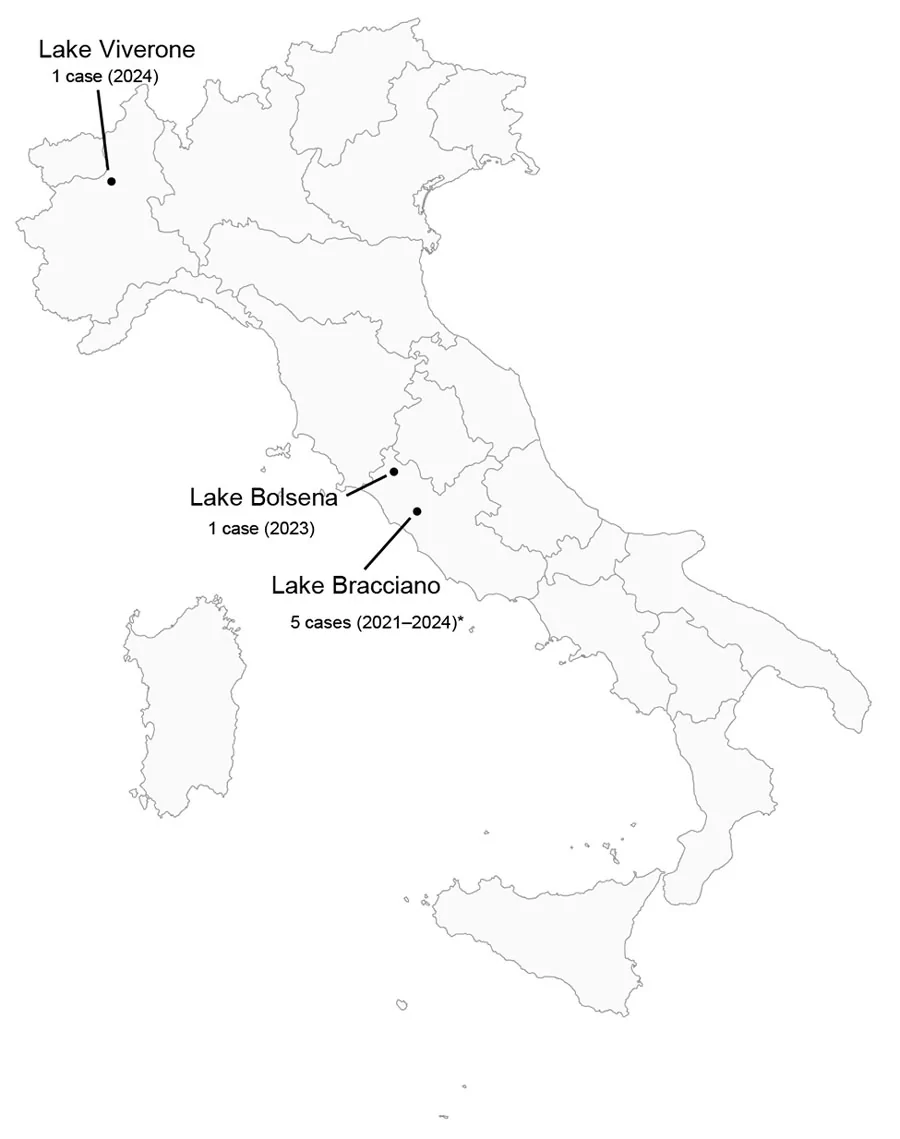
Freshwater sites associated with autochthonous canine pythiosis cases for a study of autochthonous canine pythiosis linked with freshwater exposure, Italy, 2021–2024. Dots indicate lakes linked to reported exposure histories. Labels summarize the number and year(s) of associated cases; asterisk indicates that 2 previously published cases (*6*) were included in the total count for that lake. Base map adapted from Wikimedia Commons (https://commons.wikimedia.org)

Six of the 7 infected dogs were German shepherds. Although the number of cases remains limited, this predominance is noteworthy and suggest that factors beyond environmental exposure alone might contribute to disease occurrence. Published canine series from endemic regions show variable breed distributions, suggesting that disease emergence likely reflects a multifactorial interplay between environmental exposure, host-related factors, and clinical recognition patterns. Clinical manifestations were predominantly cutaneous and characterized by chronic nodules, plaques, ulceration, and draining tracts (Figure 2); 1 dog later developed gastrointestinal involvement. Researchers have described similar cutaneous and gastrointestinal manifestations in canine pythiosis occurring in other regions of the world (*7*–*9*). The predominance of cutaneous presentations in our series may reflect route of exposure through damaged skin or recognition bias, because gastrointestinal disease may be less readily diagnosed. In several cases, the initial clinical manifestation suggested inflammatory or bacterial disorders, and empirical treatment and, in some cases, repeated surgery preceded definitive diagnosis (Appendix).

**Figure 2:**
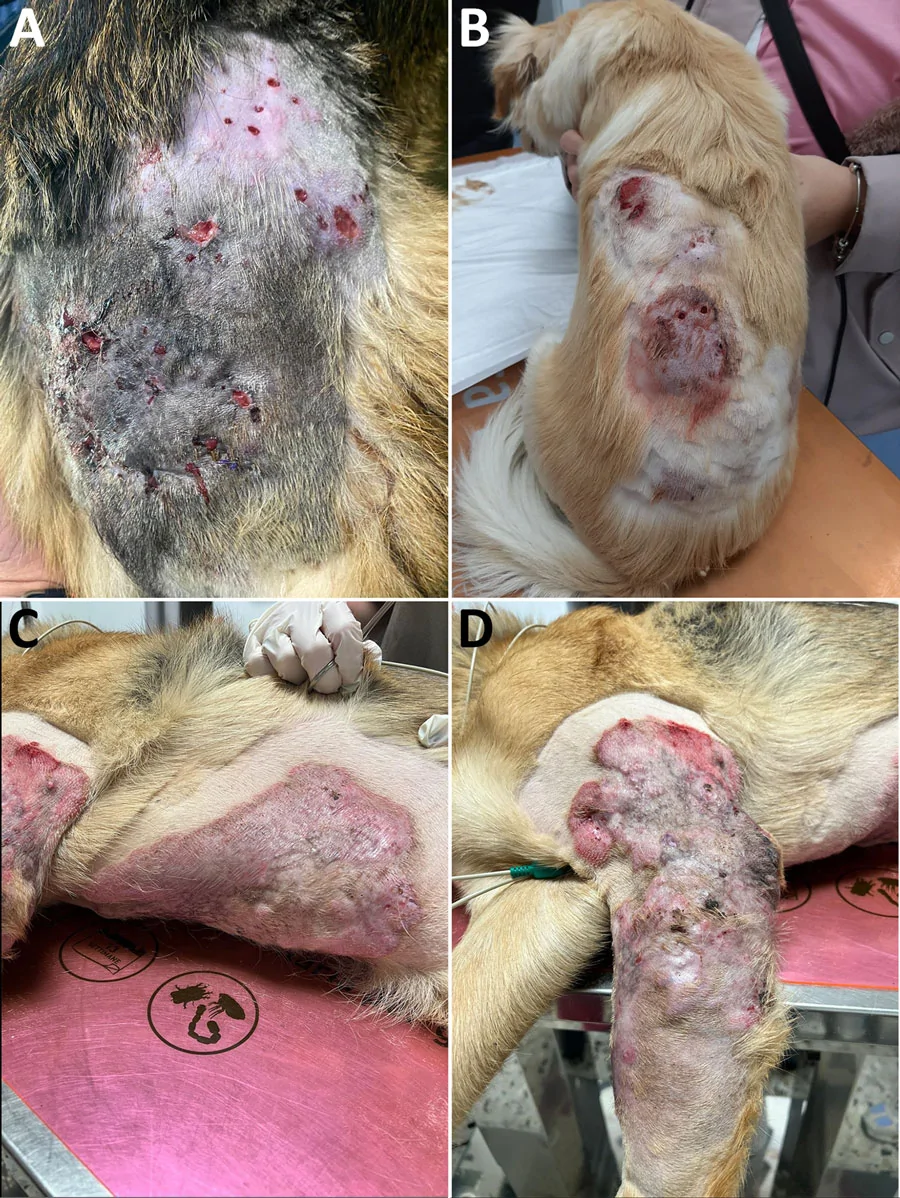
Representative cutaneous manifestations of canine pythiosis for a study of autochthonous canine pythiosis linked with freshwater exposure, Italy, 2021–2024. Lesions were characterized by chronic ulceration, nodules or plaques, draining tracts, and extensive proliferative to necrotizing cutaneous involvement. A) Extensive ulcerative and proliferative lesions involving the dorsal region. B) Ulcerative plaque affecting the dorsal thoracic region. C) Extensive ventral cutaneous involvement with ulcerative and proliferative lesions. D) Severe chronic ulcerative and exudative lesion affecting the left forelimb.

We established diagnosis by combinations of cytology, histopathology, culture, and sequencing (Appendix). Cytologic and histologic examination showed broad, irregular, sparsely septate, hyphae-like structures associated with pyogranulomatous inflammation. Internal transcribed spacer sequence data were available for 6 cases, and those isolates clustered within clade IV of the *P. insidiosum* complex, corresponding to the lineage currently designated *Pythium periculosum*. (*10*) We conducted phylogenetic analysis using representative reference sequences spanning the 4 major clades recognized within the complex (Appendix Figure). All sequences from Italy grouped within clade IV, consistent with the broader extra-American representation previously observed for this clade (*10*). One additional case, for which only large subunit sequence data were available, showed highest similarity to isolates previously assigned to the same lineage. The sequences from Italy also showed limited divergence from one another and appeared closely related within the clade IV framework, suggesting a geographically related cluster.

Our findings provide further evidence that canine pythiosis can occur in temperate settings outside traditionally recognized endemic areas. Recurrent identification of cases associated with freshwater exposure suggests that suitable environmental niches may exist in multiple regions of Italy despite the absence of classically tropical climatic conditions. From a One Health perspective, dogs may act as sentinels of environmental exposure risk relevant to multiple hosts and may contribute to recognition of previously underappreciated ecologic niches favorable to pathogenic oomycetes. Increased awareness among veterinary and human healthcare professionals will be important for earlier recognition of this emerging disease in Europe.

Appendix Additional information for autochthonous canine pythiosis linked with freshwater exposure, Italy, 2021–2024.
