# Supplementary material for: Autochthonous Canine Pythiosis Linked with Freshwater Exposure, Italy, 2021–2024
Source: Emerg Infect Dis. 2026 Sep;32(9):1526–8. doi: 10.3201/eid3209.260354 (PMC13555719; doi:10.3201/eid3209.260354)
Supplement: Supplementary file 1 [file 26-0354-Techapp-s1.pdf]

Article DOI: http://doi.org/10.3201/eid3209.260354

EID cannot ensure accessibility for supplementary materials supplied by authors. Readers who have difficulty accessing supplementary content should contact the authors for assistance.

Autochthonous Canine Pythiosis Linked with Freshwater Exposure, Italy, 2021–2024

Appendix

Detailed characteristics of recognized autochthonous canine pythiosis cases in Italy

2021 – Lake Bracciano

2-year-old female German Shepherd Dog.

Clinical presentation

Multifocal ulcerative cutaneous nodules and plaques with fistulous lesions involving multiple body sites.

Diagnostic methods

Diagnosis was based on cytology, histopathology, and ITS sequencing.

Molecular findings

ITS sequence accession number: PQ523809.

Treatment and outcome

Empirical antimicrobial therapy and surgery preceded diagnosis. Initial clinical improvement was observed after itraconazole treatment; however, the dog later developed systemic deterioration with visceral involvement and was euthanized.

2022 – Lake Bracciano (previously reported in Peano et al. 2023)

4-year-old neutered female German Shepherd Dog.

Clinical presentation

Large ulcerative cutaneous masses involved the axillary and flank regions, with an additional nodular lesion affecting a forelimb digit.

Diagnostic methods

Diagnosis was established by cytology, histopathology, and ITS sequencing.

Molecular findings

ITS sequence accession number: OQ532907.

Treatment and outcome

Multiple surgeries, antimicrobial drugs, glucocorticoids, and cyclosporine preceded diagnosis. Initial improvement after itraconazole treatment was followed by systemic progression and euthanasia.

2022 – Lake Bracciano

12-year-old spayed female German Shepherd Dog.

Clinical presentation

Progressive multifocal cutaneous lesions with draining tracts and pyogranulomatous inflammation.

Diagnostic methods

Diagnosis was based on cytology, histopathology, and LSU sequencing.

Molecular findings

LSU sequence accession number: PQ496885.

Treatment and outcome

Antimicrobial drugs, glucocorticoids, itraconazole, and terbinafine resulted in no substantial clinical improvement. The dog later died because of an unrelated acute condition.

2023 – Lake Bracciano (previously reported in Peano et al. 2023)

3-year-old male German Shepherd Dog.

Clinical presentation

Multifocal dermal and subcutaneous plaques and nodules with draining tracts and serosanguineous exudate.

Diagnostic methods

Diagnosis was established by cytology, histopathology, and ITS sequencing.

Molecular findings

ITS sequence accession number: OQ532908.

Treatment and outcome

Empirical antimicrobial drugs and corticosteroids were ineffective. Severe progressive cutaneous disease developed.

2023 – Lake Bolsena

2-year-old female mixed-breed dog.

Clinical presentation

Chronic progressive cutaneous lesions characterized by nodules, plaques, ulceration, and draining tracts. Gastrointestinal involvement developed later during disease progression.

Diagnostic methods

Diagnosis was based on cytology, fungal culture, and ITS sequencing.

Molecular findings

ITS sequence accession number: PQ525320.

Treatment and outcome

Combined prolonged treatment including itraconazole, terbinafine, antibacterial drugs, corticosteroids, and mefenoxam resulted only in transient improvement before progressive gastrointestinal involvement and euthanasia.

2024 – Lake Bracciano

4-year-old female German Shepherd Dog.

Clinical presentation

Multifocal ulcerative cutaneous lesions and draining tracts with progressive local extension.

Diagnostic methods

Diagnosis was established by cytology, fungal culture, and ITS sequencing.

Molecular findings

ITS sequence accession number: PQ525321.

Treatment and outcome

Itraconazole treatment was discontinued because of adverse effects. The dog died shortly after diagnosis.

2024 – Lake Viverone

5-year-old male German Shepherd Dog.

Clinical presentation

Progressive extensive cutaneous nodules and plaques with ulceration and chronic relapsing evolution.

Diagnostic methods

Diagnosis was based on cytology, histopathology, fungal culture, and ITS sequencing.

Molecular findings

ITS sequence accession number: PQ525322.

Treatment and outcome

Initial surgery, cephalexin, and itraconazole treatment were followed by progression to extensive cutaneous disease. Long-term treatment including terbinafine, mefenoxam, and corticosteroids resulted in clinical remission.

Diagnostic and Molecular Methods

Cytology and Histopathology

Diagnostic evaluation was performed on combinations of fine-needle aspirates, impression smears, tissue biopsies, and surgically excised specimens, depending on the individual case. Cytologic preparations were stained with rapid Romanowsky-type stains and revealed broad, irregular, sparsely septate hyphae-like structures embedded within pyogranulomatous inflammatory infiltrates.

Histopathologic examination showed granulomatous to pyogranulomatous dermatitis and panniculitis associated with irregular hyphae-like elements morphologically consistent with oomycetes. Special stains, including Grocott methenamine silver staining, were used in selected cases to enhance visualization of the organisms. In selected cases, periodic acid–Schiff staining was also performed and yielded negative results.

Culture

Culture was performed in selected cases using tissue samples or fine-needle aspirates collected from cutaneous lesions. Samples were inoculated onto Sabouraud dextrose agar and incubated at 37°C. Colonies showed rapid growth and morphologic features compatible with *Pythium* spp., including submerged or partially submerged colorless to whitish colonies with

limited aerial mycelium and irregular radiate patterns. Microscopically, hyphae appeared broad, hyaline, and sparsely septate.

Initial cultures performed on Sabouraud dextrose agar containing chloramphenicol (0,4 g/L) yielded negative results in some cases. Because chloramphenicol at this concentration may inhibit growth of *Pythium* spp., subsequent cultures were preferentially performed on unsupplemented media or on media supplemented with alternative antibacterial agents, including gentamicin and penicillin, when bacterial contamination control was required. Zoosporogenesis was not attempted.

DNA Extraction and Sequencing

Molecular identification was performed using DNA extracted either directly from clinical tissue samples or from cultures, depending on sample availability. For culture isolates, biomass was recovered from actively growing colonies; for tissue-based analyses, DNA was extracted from biopsy or surgically excised specimens collected during diagnostic procedures.

Sequencing targeted ribosomal DNA regions, including the internal transcribed spacer (ITS) region in six cases and the large subunit (LSU) ribosomal RNA gene region in one case. ITS amplification was performed using primers ITS4 and ITS5, as previously described (Peano et al., 2023). PCR products were purified and subjected to bidirectional Sanger sequencing. Consensus sequences were assembled and compared with publicly available sequences in GenBank using BLAST analysis.

One case was characterized only by LSU sequencing because only LSU sequence data were available from the original diagnostic workup. All sequences generated in this study were deposited in GenBank.

Phylogenetic Analysis

Phylogenetic analysis was performed using representative ITS reference sequences spanning the four major clades recognized within the *Pythium insidiosum* complex according to Miraglia et al. (2022). Italian canine sequences obtained in the present study, together with the two previously published Italian canine sequences (Peano et al., 2023), were aligned with reference sequences and analyzed using a maximum-likelihood approach. The tree was inferred using the Tamura–Nei model as implemented in MEGA12.

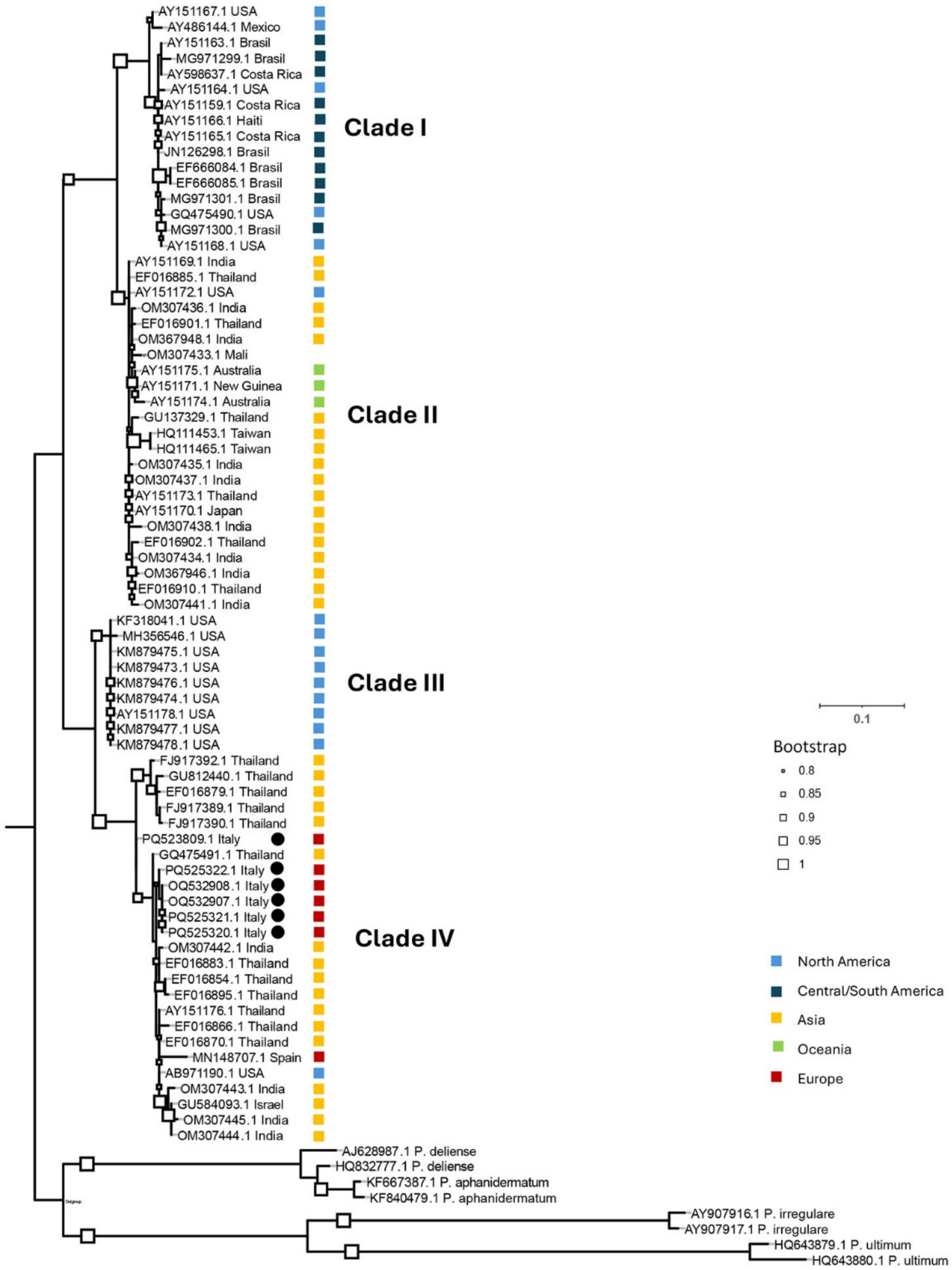

Appendix Figure. Maximum-likelihood phylogenetic tree based on internal transcribed spacer sequences of the *Pythium insidiosum* complex, including Italian canine isolates identified in a study of autochthonous canine pythiosis linked with freshwater exposure, Italy, 2021–2024. Representative reference sequences shown from the 4 major clades recognized by Miraglia et al. (https://doi.org/10.1016/j.funbio.2022.03.002). Representative *Pythium* species outside the *P. insidiosum* complex (*P. aphanidermatum*, *P. ultimum*, *P. irregulare*, and *P. deliense*) were included as outgroup taxa. Bootstrap support values ≥80% shown at corresponding nodes. Terminal labels include GenBank accession numbers and geographic origin of isolates. Colored strips indicate broad geographic regions represented in reference dataset (single African isolate left uncolored). Black circles indicate Italian canine isolates. All Italian sequences clustered within clade IV, corresponding to the lineage currently designated *Pythium periculosum*.
